# Coronavirus Proteins as Ion Channels: Current and Potential Research

**DOI:** 10.3389/fimmu.2020.573339

**Published:** 2020-10-09

**Authors:** Conor McClenaghan, Alex Hanson, Sun-Joo Lee, Colin G. Nichols

**Affiliations:** Center for Investigation of Membrane Excitability Diseases, and Department of Cell Biology and Physiology, Washington University School of Medicine, St. Louis, MO, United States

**Keywords:** Severe Acute Respiratory Syndrome coronavirus-2, ion channel, spike protein, electrophysiology, bilayer, Severe Acute Respiratory Syndrome coronavirus

## Abstract

Coronavirus (CoV) outbreaks have recently emerged as a global public health threat due to their exceptional zoonotic potential — a feature arising from their ability to infect a diverse range of potential hosts combined with their high capacity for mutation and recombination. After Severe Acute Respiratory Syndrome (SARS) CoV-1 in 2003 and Middle East Respiratory Syndrome (MERS) CoV in 2012, with the current SARS-CoV-2 pandemic we are now in the midst of the third deadly international CoV outbreak in less than 20 years. Coronavirus outbreaks present a critical threat to global public health and an urgent necessity for therapeutic options. Here, we critically examine the current evidence for ion channel activity in CoV proteins and the potential for modulation as a therapeutic approach.

## Introduction

Coronaviruses (CoVs) are enveloped, single-stranded, positive-sense RNA viruses that were first discovered in the 1930s (1). They are recognized as underlying frequent and deadly enzootic outbreaks in livestock (2), but their propensity for cross-species transmission has led to repeated human coronavirus (CoV) outbreaks, including the COVID-19 pandemic currently sweeping the world. According to the European Centre for Disease Prevention and Control, as of August 2020, Coronavirus disease 2019 (COVID−19) has caused >800,000 deaths and effective therapeutic options remain limited. Months or years may pass before successful vaccine-development efforts come to fruition, but alternative therapeutic efficacy may arise from “anti-viral” mechanisms that reduce viral fitness by interfering with stages of the viral life cycle, such as viral entry, release, assembly, and exit, or from “pro-host” mechanisms that improve host fitness by directly targeting virulence factors, thereby disrupting the fundamental origins of tissue damage and pathology. In reality, anti-viral and pro-host mechanisms may well overlap, since viral proteins often play multiple discrete functional roles that drive both pro-viral and anti-host consequences. This functional complexity points to a need for detailed understanding of underlying structure-activity-function phenomena.

Coronaviruses are spherical particles with diameter of ~125 nm enclosed in an envelope bilayer, in which the membrane (M), envelope (E) and spike (S) structural proteins are embedded (3). The S protein generates the surface spikes that mediate receptor binding and membrane fusion between the virus and the host cell, while the E and M proteins maintain the envelope shape (4). Inside the viral envelope, nucleocapsid (N) proteins stabilize the single-stranded RNA genome (4). Many studies have established the relevance of the E protein and protein 3a as fundamental pro-inflammatory SARS-CoV virulence factors (5–8), and additional studies have suggested that the inflammatory properties of E and 3a are related to their induction of ion conductances in membranes, i.e. that they are ion channel proteins (9–12). Since the initial characterization of influenza M2 (13), viral proteins which form ion channels themselves (viroporins) (14), or which can modulate host cell ion channel function (e.g. HIV-1 Vpu) (15, 16), have been reported in a variety of virus species (17), and repeatedly proposed as potential anti-viral drug targets (17–19). Some proposed viroporins are proteins with structural features that are conserved in bacterial/eukaryotic proteins, such as the viral K channel Kcv (14, 20), which contains the canonical, highly conserved, potassium-selectivity filter that is found throughout the prokaryota and eukaryota. Others include a diverse range of short peptide/proteins, typically 50 to 120 amino acids, which are predicted or have been shown to contain at least one transmembrane helix and may oligomerize to form channel-like structures with hydrophilic pores, but otherwise carry no primary structural clues to any channel nature. Ion channels in general are attractive drug-targets which account for ~15% of clinically used drugs (21). In principle the lack of homology between the proposed CoV viroporins and human ion channels provides the potential for selective modulation with small molecule or biologic therapies, and modern technologies allow for high-throughput screening of novel channel modulators (21). If major drug-discovery endeavors are to be prudent, however, convincing evidence should exist for the ion channel function of CoV proteins as well as the anti-viral effects of their functional modulation.

Although there is also a wealth of detailed functional analysis of a few viroporins, such as influenza M2 and Kcv [as reviewed in (17, 19, 22)], most putative viral channels have received relatively little electrophysiological attention, which typically requires expression of putative channels in heterologous systems or reconstitution of purified or synthesized peptides into artificial bilayers. Such approaches can be technically fickle, with important considerations for the interpretation of results, and unequivocal determination of pore-forming proteins generally requires detailed combinations of both electrophysiological analysis and structural manipulation to avoid the potential for mis-identification as a consequence of reconstitution of contaminant proteins or altered regulation of endogenous channels in heterologous expression systems (21, 23–25).

With these issues in mind, our aim is to review the relevant studies and provide a critical assessment of the current evidence for ion channel activity of SARS-CoV E protein, protein 3a, and protein 8a, and suggest potential studies to clarify existing uncertainties.

## E Protein

The envelope protein E is a small (~8–12 kDa) integral membrane protein, the biological significance of which has been comprehensively reviewed elsewhere (2). It is highly expressed in host cells during viral replication: a minor fraction is incorporated into the virion envelope, while most protein localizes in the endoplasmic reticulum, Golgi apparatus or the ER-Golgi intermediate compartment (or ERGIC) of the host cell, where CoVs bud (26, 27). E has variously been implicated in virus assembly, budding, envelope formation, virus release, inflammasome activation, and pathogenesis in different CoVs (see (2) for review), and deletion of E in recombinant viruses results in reduced viral propagation and pathogenicity (2, 28, 29). E interacts with multiple viral- and host-cell proteins and likely has multiple molecular functions (2), either in addition to, or as a consequence of, its putative role as an ion channel.

### Evidence for Ion Channel Function of E

Early reports indicated that expression of the E protein from SARS-CoV (30) and murine hepatitis virus (31), in *E. coli* or mammalian cells, could increase membrane permeability to multiple small molecules, although they did not establish whether these molecules permeated channels formed directly by E or whether expression of E increased membrane permeability *via* an indirect mechanism (31).

**Figure 1:**
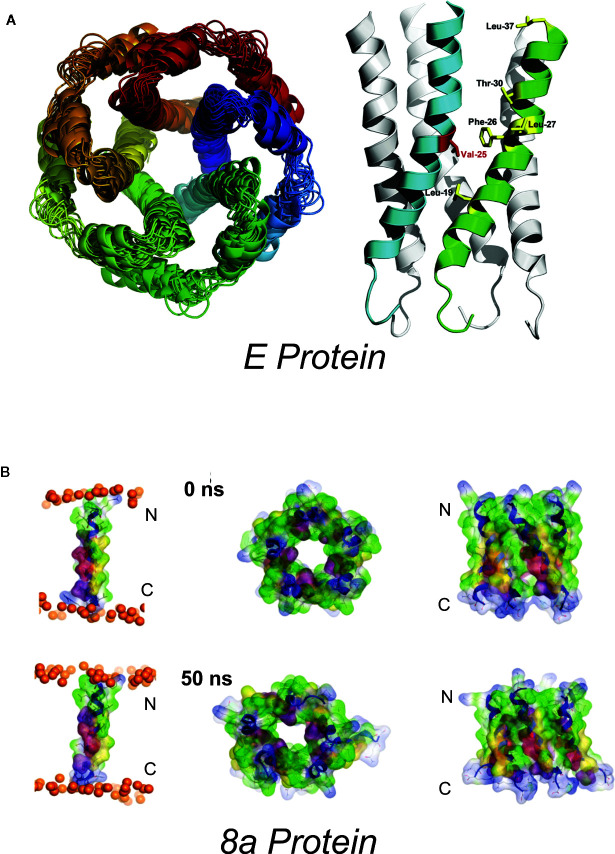
Proposed SARS-Cov E and 8a structures **(A)** Proposed homopentameric structure of the E protein (32), viewed (*left*) through the membrane, and (*right*) on the plane of the membrane. The structural model includes a ~2Å radius constriction, formed by the sidechains of V25 and V28, which could conceivably act as a channel gate, and an extended central “pore” of <6Å in radius (34) [From Surya et al. *BBA-Biomembranes* 2018 1860: 1309-1317. With publisher’s permission] **(B)**. Proposed pentameric structure of the 8a protein. (*left*) The single transmembrane domain (TMD) 8a 1–22 is shown at the beginning (0 ns) and end of a 50-ns MD simulation, (*right*). Top view (left) and side view (right) of a pentameric bundle of 8a 1–22 at the beginning (upper) and end of 50 ns MD simulation (lower). The protein backbones are drawn in blue with the side chains shown as sticks and van der Waals surface representation. Residues Thr-8, Ser-11, and 214 are shown in pink and light red, respectively. All cysteine residues, Cys-9, 213, and 217, are shown in yellow. Phosphorous atoms of the lipids are shown in orange spheres. Lipid and water molecules are omitted for clarity. [Relabeled from Hsu et al., *Proteins*. 2015; 83: 300–308. With publisher’s permission].

Western blots indicate that the E protein normally multimerizes, and a series of studies combining *in silico* and biochemical methods and NMR spectroscopy (32) provided evidence for pentameric assembly of E from SARS-CoV (33–36) and MERS (37). The structural model includes a ~2Å radius constriction, formed by the sidechains of V25 and V28, which could conceivably act as a channel gate, and an extended central “pore” of <6Å in radius (34) (**Figure 1**). The first electrophysiological characterization of E reported fluctuating currents, with indistinct gating events, and very poor signal-to-noise, when synthetic SARS-CoV E, or E proteins from HCov-229, mouse hepatitis virus (MHV) and from infectious bronchitis virus (IBV) were reconstituted into artificial bilayers (38, 39). Different apparent ion permeability series were observed: the α-CoV HCoV-229 (K^+^ > Na^+^ > Cl^−^) differed from MHV (β-CoV) and IBV (γ-CoV) (both Na^+^ > K^+^ > Cl^−^) but, in all three, selectivity inferences were based on reversal potential measurements that were confounded by very small and variable currents.

Guided by structural modeling, Torres, Liu, and colleagues engineered mutations in a short-form of SARS-CoV E (limited to the predicted transmembrane region, and flanked at both termini by 2 lysine residues to aid with solubility), and reported that N15A and V25F substitutions both altered oligomerization (2, 40), and abolished currents when proteins were reconstituted into artificial bilayers (35). Here, we have to note that the summary conductance values shown in their Figure 3 (35) are not consistent with the example traces shown. In particular 2- to 3-fold higher conductance for F23A shown in their Figure 3 is not evident in the traces provided. Curiously the double mutants V25F/A32F and N15A/V25F restored conductances that were qualitatively similar to those seen with the WT peptide. This, coupled with the additional finding that WT-associated conductances were abolished by amantadine, yet N15A/V25F-associated conductances were not, led the authors to propose that the double mutant resulted in non-specific destabilization of the membrane. That V25F/A32F currents were amantadine-insensitive and reportedly displayed no ion selectivity, whereas WT-associated conductances showed selectivity for calcium over sodium (unfortunately no drug-sensitivity data was reported for N15A/V25F double mutants), was consistent with this interpretation. Incidentally, the relevant selectivity data [their Figure 5 in (35)] was interpreted as demonstrating selectivity for sodium over calcium but the methods state that membrane voltage referred to the cis- relative to the 4 trans-bilayer chamber, and CaCl_2_ was present in the trans- chamber, so the (~20 mV) *positive* shift in the reversal potential would be consistent with a preference for Ca^2+^ to Na^+^.

The calcium permeability of SARS-CoV E was further explored by the same group, who reported that negatively-charged lipids increased the permeability ratio for calcium over chloride (11). Lipid composition also affected E-associated monovalent ion conductances, with negatively charged lipids reducing apparent unitary conductance in KCl and increasing monovalent-cation selectivity over Cl^−^, compared with neutral membranes (44, 45). Millimolar concentrations of calcium and changes in pH were reported to alter K^+^:Cl^−^ permeability ratios, suggestive of cation-interactions with negative charges in a protein-lipid complex (11). We have misgivings over the data presented in their Figure 1A, which is claimed to show 2 channel events of identical amplitude, but the example traces show the amplitudes to be obviously different. Additionally, there was apparently no increase in conductance when CaCl_2_ was increased symmetrically from ~5 mM to 300 mM (their Figure 1E), a result which seems incompatible with a Ca^2+^-permeable conductance (44, 45).

The finding that E could mediate Ca^2+^ flux in lipid membranes that mimic the high negative charge composition expected for ER and Golgi intermediate compartment (ERGIC) membranes prompted study of the potential for E to provoke abnormal calcium handling and consequent NLRP3 inflammasome activation in cells (11). Vero 6 cells transfected with cDNAs encoding the inflammasome components NLRP3, ASC and procaspase-1 and inactive pro-IL-1β secreted more active IL-1β when E was expressed. Interestingly, this increase in IL-1β secretion was attenuated when E loss-of-function mutants, N15A and V25F, were expressed, or when Ca^2+^ was removed, but there was no critical assessment of ion channel involvement, and effects of these mutations on other E functions cannot be excluded.

Additional studies have focused on the effects of the N15A and V25F mutations in SARS-CoV (10), or their equivalent mutations (T16A, A26F) in IBV (46), in infected mice or Vero E6 cells. SARS-CoV carrying the E protein N15A substitution caused markedly reduced mortality in infected mice, and a partial reversal in infection-related weight loss, whereas the introduction of the V25F mutation had little effect on either phenotype. Interestingly, it was found that mutant virus propagation in both infected mice and cultured cells was associated with the emergence of additional mutations. When synthetic E transmembrane domain peptides carrying the additional amino-acid substitutions were studied in bilayer recordings, these “revertant” mutations restored transmembrane conductances lost by the N15A or V25F substitutions. Higher mortality associated with the V25F mutant (compared to N15A) correlated with more rapid and extensive emergence of revertant mutations in V25F-mutant strains (10). Similar results were obtained in cells infected with T16A and A26F-mutant IBV (46).

In these studies, only very rudimentary electrophysiological characterization of the revertant mutants was reported, i.e. the appearance of trans-membrane currents, although certain revertant mutations (such as the A26F/K58N double and A26F/N11D/K58N triple mutants in IBV E protein) apparently result in gross changes in channel behaviour (10). Such results are broadly consistent with restoration of ion channel activity by revertant mutations recovering pathogenicity although, if channel behavior is indeed mechanistically associated with pathogenicity, then mice or cells infected with functionally very abnormal double/triple mutant channels might be expected to show altered phenotypes.

### E Ion Channel Pharmacology

Hexamethylene amiloride (HMA), an inhibitor of the HIV-1 Vpu virus ion channel, was reported to inhibit HCOV-229 and MHV E, but not IBV E (which was, if anything, apparently activated) (39). A single dose-response experiment returned an IC50 <10 μM, consistent with potencies observed in plaque formation assays showing HMA inhibition of virus plaque growth for MHV. The authors argued that lack of HMA effect on plaque growth in cells infected with MHV virus in which E had been deleted (MHV ΔE) points toward an E-mediated effect of HMA. However, from the examples shown, HMA-treated cells infected with MHV ΔE appear to exhibit smaller plaques than cells treated with amiloride, which was without effect on E conductances, arguing against a causative effect of ion channel conductance block (39). A useful further comparison would be to determine the effect of HMA on IBV fitness, since IBV E was reported to be insensitive to HMA and thus no effect of the drug on IBV would be expected if the drug works *via* E viroporin inhibition. In separate studies, amantadine was claimed to bind to, and inhibit, SARS-CoV E based on surface plasmon resonance and bilayer electrophysiology (35).

### Potential Future Studies Into E Ion Channel Function

Reports of very different ion channel properties for E proteins of various different coronaviruses and, conversely, for the same protein in different experiments, raises concerns that some results may be artefactual. Of note, example bilayer recordings in some reports look strikingly different from other reported E viroporin conductances, with long-lived open states in both neutral and negatively charged lipid bilayers (9, 11, 44, 45), contrasting with noisy, fluctuating conductances previously reported (35, 38, 39). The reason for such variable channel-gating behavior is not immediately apparent. Of major concern, there is clear reproduction of the identical example trace (with edited scale bars) being attributed to E in one publication [**Figure 3A** (45)] and the SARS-CoV 3a protein in another [**Figure 3A** (9)]. Expression of SARS-CoV E in cellular membranes after transient transfection of HEK293 cells (34) provides one potentially useful approach for more detailed electrophysiological characterization, and for confirmation of findings from synthesized peptides in artificial bilayers. With consideration of the published reports of lipid sensitivity (44, 45), properties intrinsic to the protein would be expected to be conserved across different experimental systems. One essential caveat to such an approach is of course the possibility of modulation of endogenous proteins, although acute inhibition of currents with anti-E antibodies, as originally reported by Wilson and colleagues (38, 39), could provide evidence of at least close proximity and direct regulation.

Further experiments to determine ion selectivity could involve substitution of both physiological cations and anions with larger ions, and could be interpreted alongside the suggested NMR structures to define the properties of the channel pore and the relative contributions of ion radius and hydration on permeation. Building on the reported differences in selectivity for α-CoV (HCoV-229) vs β-CoV (MHV) and γ-CoV (IBV), a detailed selectivity series analysis combined with chimeric or mutational approaches may help to determine which parts of the proteins contribute to selectivity specifically, and how different E proteins support distinct selectivity. Additional insights to E channel function might be provided by studies of channel gating combined with mutagenesis, guided by, and iteratively refining, structural models. Further, electrophysiological study could dissect the mechanism of amantadine and HMA inhibition, by including investigations of voltage-dependency of inhibition and mutational scans of potential binding sites. Again, comparison of HMA-sensitive E from HCOV-229 and MHV, versus HMA-insensitive IBV, could reveal the basis for differential binding and/or inhibition. Optimization of E expression in mammalian cells could also facilitate high-throughput drug screening using automated patch clamp or fluorescent indicators to screen for both inhibitors or positive-modulators to generate experimental therapeutics or key pharmacological tool compounds. Cross-validation of any findings in cell lines by repetition in reductionist bilayer experiments could confirm direct effects. Extrapolating findings from heterologous systems to *in vivo* function is complicated by the potential for function to vary depending on the cellular localization of the protein, which must always be considered.

### 3a Protein

**Figure 2:**
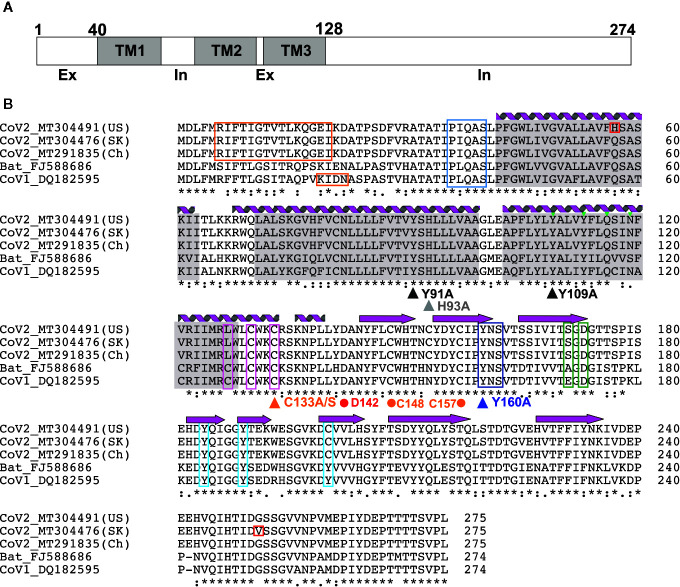
3a Protein topology and sequence alignment among corona viruses **(A)** 3a protein topology. **(B)** Multiple sequence alignment of 3a protein from corona viruses. Secondary structures [alpha helices (coils) and beta strands (arrows)] observed in the EM structure by Kern et al. (41) are indicated. Transmembrane regions (gray), novel mutations in CoV-2 (red), TRAF-binding motif (blue), epitopes for natural antibodies against 3A (orange), cysteines involved in dimer formation (magenta), internalization signal (purple), ER trafficking motif (green) and caveolin binding motif (cyan) are shown. Triangles indicate mutations suggested to affect 3a ion channel activity; dots indicate potentially critical residues inferred from the new EM structure.

The 3a protein [ORF3 (47), X1 (48), ORF3a (49) and U274 (50)] is encoded by a gene located between the S and E genes within the SNE (S neighbor E) locus in SARS-CoV genomes (48). The majority of investigations into 3a function have focused on the SARS-CoV protein. 3a from SARS-CoV-2, the virus responsible for the COVID-19 pandemic, is more closely related to Bat coronaviruses than SARS-CoV, although high sequence similarity is maintained in critical regions (**Figure 2**) between SARS-Cov and SARS-CoV-2. In particular, TM-helix polar residues are well conserved, as is C133 which is critical for oligomerization (see below), suggesting that the 3a proteins of SARS-CoV-2 and SARS-CoV may have similar molecular function and roles.

Heterologous expression in *E. coli*, as well as subsequent immunohistochemistry of commercially prepared slides of SARS‐CoV‐infected cells, revealed a *bona fide* protein product in infected host cells (51). Subsequent studies have shown that 3a predominantly localizes to the Golgi apparatus and plasma membrane (51), as well as other subcellular organelles, including endosomes and lysosomes, but rarely in ER or mitochondria (49, 50). While first proposed to be non-structural, later reports have suggested that 3a incorporates into virus particles and directly interacts with the Spike (S), Membrane (M) and E structural proteins and the accessory protein orf7a (52–54). 3a has drawn major attention as a potential SARS-CoV therapeutic target prompted by the finding that 40-50% of convalescent SARS patients developed antibodies against 3a N-terminal peptides (55–57). One study reported that serum raised against the N-terminal peptide showed a SARS-CoV neutralizing effect (58) although another study showed no neutralizing effect by mouse serum immunized by the 3a ectodomain peptide (55). Positive selection was observed in 3a along with S during the SARS outbreak in 2003, suggesting that the 3a protein may play a critical role in adaptation to new environments and hence virus survival (59). In the current COVID-19 pandemic, phylogenetic analysis has identified three major lineages of SARS-CoV-2 throughout the world, revealing a non-synonymous mutation (G26144T; G251V) in the cytoplasmic domain of 3a that is more prevalent in Europe and the US, and that distinguishes the third group (60). No biological consequence of the mutation has been established, but the fact that the G251 residue is otherwise well conserved in SARS-CoV and SARS-CoV-2 may reflect the importance of this residue and suggest that variation may alter 3a function (**Figure 2**).

Roles of 3a in viral pathogenesis, virulence and disease outcome have been extensively studied in both *in vitro* cell cultures and *in vivo* in mice and *Drosophila*. Although the 3a gene-deleted recombinant virus, SARS-CoV ΔORF3a, replicated with slightly reduced efficiency *in vitro* and at a comparable level to wild type viruses in mice (12, 61, 62), 3a expression in cultured cells led to cytopathic effects (12, 63, 64) through both apoptotic (62, 65, 66) and necrotic cell death pathways (12, 63), and affected egress of virus from host cells. These results indicate that 3a is not essential for virus particle formation but may affect virus packaging and release. As an excellent in-depth review of 3a can be found elsewhere (67), we limit our discussion to 3a protein structure and its potential ion channel function.

### 3a Protein Structures

**Figure 3:**
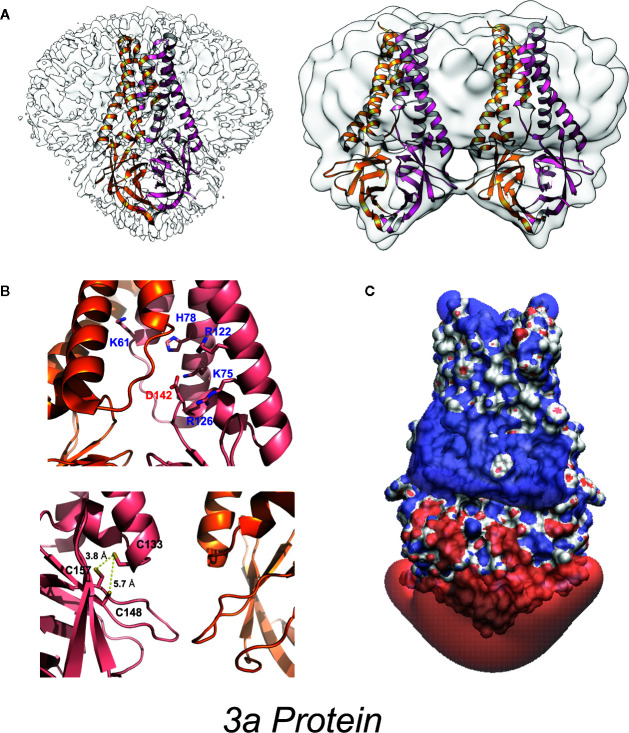
High-resolution 3a protein structure **(A)** Model of 3a dimer (left) and dimer-of-dimer (right) proteins embedded in lipid nanodiscs (PDB: 6XDC) (41). **(B)** *(above)* Location of charged residues within the cavity. (*below*) Location of cysteine residues near the dimer-dimer interface. **(C)** Space-filling model colored to illustrate the isoelectric potential of the dimeric protein (+3 blue and −3 red) computed by PDB2PQR (42) and APBS (43) webservers with default settings.

3a is a 31-kDa protein composed of an N-terminal ectodomain (amino acids 1-39), three transmembrane helices (TM1, TM2, TM3;40-128) and a cytoplasmic domain (129-274) (**Figures 2**, **3**). Subunit association studies suggested that the proteins assemble as homo-tetramers in a dimer-of-dimer configuration (68), that is strictly dependent on the presence of C133 (68). Kern et al. recently determined near-atomic resolution (2.8 Å) single particle cryo-EM structures of the 3a protomer (dimer) as well as a tetrameric (dimer-of-dimer) form (41). As expected from the absence of sequence homology to any other proteins of known structures, 3a protein adopted a novel fold with three trans-membrane spanning helices, a cytoplasmic domain consisting predominantly of two beta sheets, and a short alpha helix connecting the two domains (**Figure 3**). Both transmembrane and cytoplasmic domains from one subunit form extensive interactions with the other subunit in the protomer. Potential ion permeation pathways reside in each dimer; hydrophobic bifurcated paths with a tight constriction in the extracellular half (potentially consistent with the channel being in a non-conductive state) are connected to a wide inner cavity near the cytoplasmic end with two side openings to the environment; one within a subunit between TM2 and TM3 with hydrophobic residues lining the entrance and the other at the subunit interface (TM1 and TM3) with polar and charged residues lining the surface. An aspartic acid (D142) located at the apex of the short alpha helix connecting the transmembrane and cytoplasmic domain provides a single negatively charged pore-lining residue between the inner cavity and the side opening. These structural features suggest that the side opening at the subunit interface may be the path for water and ion movements. The low resolution (6.5 Å) electron density map of tetramers reveals the side-by-side assembly of two protomers. Interestingly C133, required for oligomeric assembly, is located near the protomer interface without directly facing the interface, in close proximity to two other cysteines (C148 and C157), potentially close enough to form disulfide bonds that may affect oligomer stabilities (**Figure 3**).

### 3a Ion Channel Activity

3a was first claimed to be a K^+^ selective channel based on experiments showing the presence of Ba^2+^-sensitive K^+^ currents in transfected HEK293 cells (62), and in *Xenopus* oocytes injected with 3a cRNA (68). Appropriate shifts of K^+^ reversal potentials with asymmetric elevations in ion concentration corroborated K^+^ selectivity of the channel activity (18, 68–70). This correlation between two different expression systems strengthens the argument that 3a expression leads to appearance of a K^+^ conductance but, as noted above, such experiments cannot trivially exclude the possibility that the 3a protein activates an endogenous channel. For most ion channels, single-channel conductances provide a molecular “signature” that is unique and hence can define currents from endogenous or contaminant channels in recombinant expression systems. Unfortunately, while 3a expressed in oocyte membranes resulted in the appearance of a mild outwardly-rectifying single channel conductance, with a 90-pS slope conductance at positive potentials (in 100 mM KCl), strikingly different and varying properties were reported for 3a protein in bilayer experiments, including currents with maximum conductance ~12 pS in symmetrical 500 mM KCl) and maximum conductance ~56 pS in symmetrical 500 mM CaCl_2_ (71). A ten-fold KCl gradient (500:50 mM, cis:trans) in the same study resulted in a ~15 mV shift in the reversal potential, indicative of a mild Cl^−^ preference (71). Anion-replacement or large cation-replacement experiments would have been useful to rule out non-specific conductances through bilayer perturbation and demonstrate whether there was any true selectivity for different ion species. In sharp contrast, another study purified 3a from High-Five insect cells and performed single channel recordings in artificial bilayers, claiming to show a mild selectivity for monovalent cations (9), although, as noted, this was presented with a:representative trace” that had been attributed as a SARS-CoV E channel current in a previous publication (9, 45), seriously questioning the validity of these studies. Kern et al. have now functionally characterized the dimeric 3a proteins after reconstitution into synthetic liposomes (41). They analyzed reversal potential shifts in bi-ionic conditions to predict permeability ratios (P_X_/P_K+_): Ca^2+^ ~2 > K^+^ ~1 > Na^+^ ~0.6 > NMDG^+^ ~0.3, which is presumably based on the reported voltages referring to the pipette relative to the bath electrode (i.e. inverted from standard electrophysiological convention). The data indicate that reconstituted 3a protein generates a non-selective cationic channel potentially with a large pore (to accommodate NMDG^+^) and high single channel conductance (375 pS at – 80 mV). Permeation of large ions would likely require significant conformational changes from the resolved structure, given its narrow minimum pore radius. Of note is that single channel properties of 3a proteins were highly dependent on the permeating ions in particular with substantial flickering with Ca^2+^ and much smaller unitary conductances with NMDG^+^, Ca^2+^, and Na^+^ than with K^+^ ions, which suggests that the protein may undergo permeant ion-dependent conformational changes. Unexplained is how cation selectivity might arise: the cytoplasmic sites through which ion and water are proposed to enter the permeation pathways all exhibit positive potential (**Figure 3**); the inner cavity holds 5 basic residues per subunit while D142 is the only acidic residue in that space (**Figure 3**).

### Modulation of 3a Ion Channel Function and Virus Pathogenicity

Overexpression of 3a in eukaryotic cells induces apoptosis, manifested in nuclear condensation, caspase-8 and -9 activity, increased cytosolic cytochrome C signaling in cultured cells and the development of a “rough-eye” phenotype associated with apoptosis in *Drosophila* (62). In the presence of the potassium channel inhibitors 4-AP and Ba^2+^, or when the C133A/Y160A mutations were included, apoptotic markers were significantly reduced. However, channel inhibitors or mutations only resulted in partial reversal of pathology, which was not quantitatively matched to the complete loss of K^+^ currents (62), consistent with 3a-driven apoptosis being at most only partly dependent on channel activity. Overexpression of 3a also causes necrotic host cell death with concurrent secretions of pro-inflammatory cytokines (12, 63), but the triple C127S/C130S/C133S mutation, which abolishes ion channel function and reduces tetramerization, did not affect pro-inflammatory IL-8 production (12). In addition, both WT 3a and the C133A mutant provoked similar NLR family pyrin domain containing 3 (NLRP3) inflammasome activation (63). These results indicate that ion channel activity of 3a protein *per se* may not be critical for triggering necrotic cell death.

Two molecules identified from traditional Chinese medicines have been reported to inhibit 3a K^+^ conduction. Early studies indicated that Emodin, an anthraquinone compound first identified with an inhibitory effect on S protein interaction with angiotensin converting enzyme 2 (ACE2) (72) both inhibited 3a K^+^ conduction and half inhibited virus release at ~20 µM (69). Juglanin, a kaempferol glycoside, also completely blocked 3a-mediated current at 10 µM (69), but its influence on viral release was not tested. The recent study by Kern et al. showed that Emodin did not affect purified 3a channel activity, and no Emodin electron density was detected in their single particle cryo EM trial of 3a proteins in the presence of the compound (41). These results might imply that emodin effects on 3a proteins observed in cells is indirect.

### Potential Future Studies of 3a Electrophysiology

Recombinant expression in eukaryotic cells provided evidence that 3a generates K^+^-selective channels, whereas bilayer recordings suggest that 3a generates relatively cation non-selective channels. Ion selectivity is likely to be an intrinsic property of any channel, and such a discrepancy between cellular and bilayer studies is disconcerting. Moreover, ion selectivity is the key determinant of the functional relevance of any ion channel, and without clarity on this, any role for ion channel activity of 3a in virus biology will remain unclear. While the studies by Kern et al. strongly suggest that 3a proteins are indeed non-selective cation channels, Ba^2+^ sensitivity of the purified and reconstituted 3a proteins has not been examined. This could be a critical test of whether Ba^2+^ sensitive currents observed in 3a transfected eukaryotic cells are carried by 3a or by other K channels whose expression might have been augmented by 3a proteins. If the latter turns out to be the case, just how Ba^2+^ sensitive potassium currents are induced by 3a proteins would be of particular importance.

### 4a Protein

HCoV-229E is generally considered a relatively benign human coronavirus responsible for the common cold, although a recent case report documenting an infection associated with ARDS suggests a potentially more dangerous pathology (73). The HCoV-229E genome includes Orf4a (4a) which encodes a short (133 AA) accessory protein with some limited homology (17% identity) with SARS-CoV 3a, and transmembrane domain prediction programs have been used to suggest that 4a shares the 3 TMD architecture reported for 3a (74). When 4a was expressed in HCoV-229E infected Huh-7 cells, tagged proteins were shown to localize with a marker of the endoplasmic reticulum/Golgi intermediate compartment (74), as previously reported elsewhere for SARS-CoV 3a (51, 75) and HCoV-NH63 ORF3 (76). Intriguingly, 4a expression also rescued survival in a potassium-transporter deficient yeast strain (74), good evidence that 4a either mediates K^+^ transport directly, or activates an endogenous transport mechanism. In contrast to 3a, expression of 4a in *Xenopus* oocytes resulted in small currents that exhibited distinctive time-dependent activation at negative voltages. Although the authors proposed these were cation currents, they are reminiscent of endogenous Cl^−^ conductances that have previously been reported following expression of multiple influenza virus proteins in oocytes (77). Anion substitution experiments, which might have ruled out this possibility, were not performed. Although cation substitution experiments appeared to show some selectivity for Li^+^, Na^+^ and Cs^+^ over K^+^ or Rb+, the magnitude of the conductances were too small for reliable assessment of reversal potential. Permeation of larger cations could be informative but, again, mutational analyses to probe specific channel properties are necessary to definitively test the ion channel function of 4a.

### 8a Protein

The hydrophobic 39 AA SARS-CoV 8a peptide came into being following a remarkable genetic event, apparently occurring shortly after zoonotic transmission to humans, in which a 29-nt deletion split the ORF8 gene (which encodes the full-length 8ab^+^ protein found in animals and early human isolates) into two distinct open reading frames, resulting in 8a and 8b peptides (78). Antibodies against 8a have been found in a small subset of SARS patients, suggesting the protein is expressed in infected humans. 8a has been implicated in viral replication and the induction of apoptosis, and has been reported to localize within mitochondria or within the endoplasmic reticulum (79, 80). However, a recent study using mouse-adapted (MA15) recombinant SARS showed no major effect of 8a deletion on virus titer in Vero E6 cells, virus growth *in vivo* in BALB/c mice, or mouse survival (9). Chen et al. (81) used *in silico* prediction of transmembrane topology and molecular dynamics simulations to propose multiple potential oligomultimers, including tetrameric-, pentameric-, and hexameric channel-like complexes, with hydrated pores lined by serine, threonine and cysteine residues (**Figure 1**). Subsequent MD simulations of pentameric 8a complexes were used for potential of mean force (PMF) calculations for Na^+^, K^+^, Cl^−^, and Ca^2+^ ions along the predicted permeation pathway (82). Similar peak PMF energy values around 2 kcal/mol were observed for all ions tested, yet Cl^−^ ions permeated more readily under applied voltages — an effect attributed to a voltage-dependent widening of the pore during 50 ns permeation simulations which was not present in the brief PMF calculations. Experimental characterization of 8a has been limited to a single electrophysiological study of synthesized 8a peptide reconstituted into artificial lipid bilayers (81). Representative traces show noisy channel-like events and Ohmic behavior with low (~9 pS) conductance in symmetrical 300 mM KCl solutions. A ten-fold asymmetric elevation in ion concentration in the trans-chamber of the bilayer set-up resulted in an ~+30 mV shift in the reversal potential, which implies a weak cation-selectivity, in apparent conflict with the MD permeation studies and with the minor differences in peak energy barrier for cations and anions in PMF calculations (82). Unfortunately, the study again raises questions regarding overall interpretations: for example, it is not clear how the authors could reliably resolve the mean conductance in asymmetric ionic conditions given the tiny currents at the voltage cited (their Figure 2E).

Additional electrophysiological studies are needed to establish whether 8a indeed forms an ion channel, and there is currently no evidence that modulation of putative ion channel activity of 8a leads to anti-viral and/or pro-host effects. Predictions from *in silico* modeling of voltage-dependent changes in the narrowest region of the pore (from N-terminal side at 0 mV to the C-terminal side at 45 mV), an overall decrease in the minimal pore radius with increasing voltage, and a specific increase in the observed Cl^−^ permeation at positive voltages may point toward voltage-dependent changes in ion selectivity, conductance, or open probability which could be tested by further electrophysiological analysis. Furthermore, the relatively small minimum pore radius proposed (81, 82) suggests that permeant ion substitution with large ions (such as NMDG, quaternary ammonium ions, gluconate) should lead to marked decrease in conductance or channel block. For 8a, as for all other putative viroporins, mutagenesis studies, guided by predicted structures and involving alterations of pore electrostatics, alongside detailed ion substitution experiments in multiple cell types (to mitigate the confounding effects of endogenous channels) could provide clear-cut evidence of *bona fide* ion channel behavior.

## Concluding Remarks

In conclusion, despite a large number of reports, definitive studies regarding SARS-CoV viroporin activity remain few, and the strength of evidence for *bona fide* ion channel behavior of any SARS-CoV protein has until now been limited. It is not trivial to unequivocally demonstrate intrinsic ion channel activity of a protein but the field would benefit from detailed, parallel studies of channel behaviors in multiple systems. Identification of mutations that specifically modify channel properties (i.e. gating, selectivity) provides the gold standard for such studies, but is currently absent from the SARS-CoV field. The recent study of Kern et al. provides a high-resolution structure of 3a and compelling evidence of channel activity, although, at the time of writing, this study is still only published in pre-print form. Even when ion channel activity is convincingly demonstrated, determining functional relevance is of course difficult. This may be especially true for viruses, where a single viral protein may exhibit several discrete activities that do not translate predictably to pathogenic functions

## Author Contributions

All authors contributed equally to writing and editing the manuscript. All authors contributed to the article and approved the submitted version.

## Funding

Our own work is supported by NIH R35 HL140024 to CN, and K99 HL150277 to CM. Fellowship support of AH is provided by NIH T32 HL125241.

## Conflict of Interest

The authors declare that the research was conducted in the absence of any commercial or financial relationships that could be construed as a potential conflict of interest.
